# Parkinson’s Disease-Related Genes and Lipid Alteration

**DOI:** 10.3390/ijms22147630

**Published:** 2021-07-16

**Authors:** Milena Fais, Antonio Dore, Manuela Galioto, Grazia Galleri, Claudia Crosio, Ciro Iaccarino

**Affiliations:** 1 Department of Biomedical Sciences, University of Sassari, 07100 Sassari, Italy; faismilena@gmail.com (M.F.); galioto@uniss.it (M.G.); ccrosio@uniss.it (C.C.); 2 Istituto di Scienza delle Produzioni Alimentari, CNR, 07040 Sassari, Italy; antonio.dore@cnr.it; 3 Department of Medical, Surgical and Experimental Sciences, University of Sassari, 07100 Sassari, Italy; galleri@uniss.it

**Keywords:** Parkinson’s disease, lipid metabolism, alpha-synuclein, GBA, LRRK2

## Abstract

Parkinson’s disease (PD) is a complex and progressive neurodegenerative disorder with a prevalence of approximately 0.5–1% among those aged 65–70 years. Although most of its clinical manifestations are due to a loss of dopaminergic neurons, the PD etiology is largely unknown. PD is caused by a combination of genetic and environmental factors, and the exact interplay between genes and the environment is still debated. Several biological processes have been implicated in PD, including mitochondrial or lysosomal dysfunctions, alteration in protein clearance, and neuroinflammation, but a common molecular mechanism connecting the different cellular alterations remains incompletely understood. Accumulating evidence underlines a significant role of lipids in the pathological pathways leading to PD. Beside the well-described lipid alteration in idiopathic PD, this review summarizes the several lipid alterations observed in experimental models expressing PD-related genes and suggests a possible scenario in relationship to the molecular mechanisms of neuronal toxicity. PD could be considered a lipid-induced proteinopathy, where alteration in lipid composition or metabolism could induce protein alteration—for instance, alpha-synuclein accumulation—and finally neuronal death.

## 1. Introduction

Parkinson’s Disease (PD) is the second most common neurodegenerative disorder characterized mainly by the progressive loss of dopaminergic neurons of the substantia nigra pars compacta and other monoaminergic cell groups in the brainstem [1]. The depletion of the neurotransmitter dopamine in the nigrostriatal system leads to the hallmark motor symptoms of PD, such as bradykinesia, rigidity, resting tremor, and postural and gait impairment. Μore than 6 million people worldwide are living with PD and this number is estimated to double by the year 2040. Importantly, PD symptoms appear when more than 50–70% of nigrostriatal dopaminergic neurons have been lost; thus, the human population of undiagnosed asymptomatic patients is probably large. Up to now, no treatment can slow the dopaminergic neuronal death and PD progression of PD; the main pharmacological treatments (levodopa and dopamine agonists) only relieve motor symptoms. Neuropathologically, PD is characterized by the presence of proteinaceous inclusions termed Lewy bodies (LBs), primary composed of alpha-synuclein (α-syn) aggregates. The etiology of PD is unknown, although older age and neurotoxins are established risk factors, and smoking appears to be protective [1]. Although the pathogenesis of PD remains incompletely understood, both genetic susceptibility and environmental factors appear to be involved [2]. The identification of rare familial forms of parkinsonism and the subsequent cloning of causal genetic mutations has had a significant impact on our understanding of the molecular mechanisms underlying idiopathic PD. Genes whose mutations have been associated with parkinsonism include autosomal dominantly (α-synuclein, LRRK2, VPS35, EIF4G1) as well as recessively (PARK2, PINK1, DJ-1, SYNJ1, and PLA2G6) inherited mutations [3,4]. Moreover, heterozygous mutations in the gene encoding β-glucocerebrosidase (GBA) are considered the greatest genetic risk factor for developing PD [5].

The similarities in the pathological and clinical phenotypes between sporadic and familiar PD forms in humans suggest that different causes may lead to a common neuropathological cascade of events. In this context, lipids are implicated in many aspects of PD pathology, ranging from specific cytotoxic interactions with PD-causative genes, lipid pathways or metabolism alterations in PD patients or experimental models to mutations in enzymes involved in lipid metabolism that significantly enhance PD risk. Different reviews exploring lipid alteration in idiopathic PD have already been published [6,7], while an extensive analysis of significant alteration on either lipid pathways or metabolism in familial PD patients or in experimental models expressing different PD-causative or -susceptibility genes is largely lacking.

## 2. Lipid Alteration in the Different PD-Related Genes

### 2.1. Alpha-Synuclein (PARK1-4)

Different lines of evidence underline an important role of lipids in α-syn physiology and pathology. Likewise, α-syn possibly regulates lipid metabolism. Alpha-syn has a disordered conformation in solution, while it can assume an α-helical structure upon membrane lipid binding [8]. The lipid composition strongly affects α-syn binding to membranes as well as α-syn aggregation and propagation. Initially, Davidson and colleagues showed that α-syn preferentially binds to vesicles containing acidic phospholipids [8], then the results were confirmed by other independent groups [9,10]. Moreover, either lipid composition, for instance the presence of 1-O-hexadecyl-2-acetyl-sn-glycero-3-phosphocholine (C16:0 PAF) [11] or arachidonoyl and docosahexaenoyl polyunsaturated fatty acids [12] or changes in chemical properties of the lipids [13] are likely to be key factors in regulating the balance between functional and deleterious interactions of α-syn with membranes. Docosahexaenoic acid (DHA), an abundant fatty acid of neuronal membranes, readily promotes α-syn aggregation and the morphology of aggregates is dependent on the ratio between the protein content and DHA [14]. Physiologically, α-syn was first shown to co-localize with synaptic vesicles [15] and afterwards, it was identified around different membrane structures [16] and brain lipids [17]. For instance, α-syn is associated to phospholipid monolayers, mainly containing high levels of triacylglycerols (TAGs), around lipid droplets (LD), and, interestingly, the pathological synuclein mutant seems to affect the TAGs hydrolysis compared with WT α-syn [18]. Moreover, α-syn is associated to mitochondria and this interaction requires cardiolipin, a mitochondria-specific lipid, and the anionic charge of the diphosphatidyl glycerol headgroup in particular [10]. Cole at al. demonstrated that cytosolic acidification rapidly induces α-syn translocation to mitochondrial surface, likely by low pH-induced exposure of cardiolipin on the mitochondrial membrane [19].

An interesting recent study, mostly based on correlative light and electron microscopy, postulated that LBs are largely composed of lipids, membrane fragments, and membranous organelles, such as vesicles [20]. This would be consistent with the idea that α-syn–membrane interaction could be the nucleation event in the aggregation of the α-syn protein [21]. Importantly, the prospect that at least some LBs may be largely composed of vesicle clusters, lipid droplets, membranes, and mitochondria rather than solely fibrillar α-syn offers a potential important change in the way we conceptualize PD pathogenesis.

As mentioned above, α-syn also plays an important role in lipid metabolism and homeostasis. Alpha-syn expression regulates the acyl-CoA synthetase activity, likely modulating the acyl-CoA synthetase localization to endoplasmic reticulum leading to an alteration in arachidonate (20:4n–6) turnover in brain phospholipids in α-syn KO mice [22]. Likewise, activation of Acyl-CoA synthetase leading to an increase in TAG content was obtained by A53T α-syn overexpression in N27 dopaminergic neuronal cells [23]. Controversial results have been accumulated related to alpha-synuclein inhibition of phospholipase D activity. Although initial studies have identified alpha and beta-synucleins as potent and specific inhibitors of phospholipase D2 activity [24], subsequent studies, using either purified proteins in cell-free assays or different cell line experimental models, have not confirmed a significant inhibitory effect of α-syn on the PLD activity [25]. However, many experimental results strongly suggest a functional interaction between α-syn and phospholipase D. For instance, pld2 overexpression in rat Substantia Nigra pars compacta causes the loss of dopaminergic neurons due to excess of lipase activity, and, interestingly, α-syn co-expression suppressed the PLD2 toxicity [26]. Moreover, phospholipase D1 regulates the autophagic flux and clearance of alpha-synuclein aggregates [27], while the overexpression of wild-type α-syn in human neuroblastoma cells inhibits the PLD1 expression [28].

The analysis of brain phospholipids in alpha-syn knockout mice has shown different alteration in lipid metabolism with an increase in docosahexaenoic acid incorporation and turnover [29] or an increase in cholesteryl esters and cholesterol mass [30,31] and a reduction in both cardiolipin and its precursor phosphatidylglycerol concentration [32].

### 2.2. Glucocerebrosidase (GBA)

Heterozygous mutations in the gba gene, encoding lysosomal enzyme glucocerebrosidase (GCase), represent the most common genetic risk factor for PD. GBA deficiency, first discovered in patients suffering from Gaucher Disease (GD), results in the accumulation of glycolipids in macrophages residing in liver, lung and spleen and importantly, type 2 and type 3 GD also exhibit deficits involving the central nervous system [33]. GBA mutations occur in 7–15% of PD cases [5,34], and the PD risk increase is between 3-fold for carriers of mild GBA mutation (N370S) and 15-fold for carriers of more severe GBA mutation (L444P or 84GG) [35]. Between the different genes related to PD, GBA is clearly and directly implicated in lipid metabolism. Up to now, different heterozygous GBA mutations associated with PD have been identified: N370S, L444P, and E326K. Pathological GBA mutations result in reducted glucocerebrosidase activity [36], and, importantly, PD patients without GBA mutations also exhibit lower levels of GCase activity in the central nervous system, further confirming the contribution of the gba gene to the disease pathogenesis [37,38].

The exact mechanism of GBA mutation toxicity is still debated; both loss- and gain-of-function hypotheses are supported by experimental evidence, and these hypotheses are not mutually exclusive. Generation of GBA animal models has permitted a more extensive analysis of GBA-related molecular mechanisms leading to cell toxicity. In mice, the presence of pathological point mutations in homozygotes leads to strong decrease in GCase activity in liver, lung, and spleen, with a residual activity between 2% and 25% compared with WT [39], while heterozygous mice bearing the L444P mutation show a 40% reduction in GCase activity [40]. In the serum of PD patients carrying pathological GBA mutations, a significant modification in lipid composition has been described: monohexosylceramide, ceramide, and sphingomyelin were elevated, while phosphatidic acid, phosphatidylethanolamine, plasmalogen phosphatidylethanolamine, and acyl phosphatidylglycerol were decreased [41]. The exact mechanism by which GCase dysfunction increases the risk of PD remains elusive, although a number of different pathological mechanisms have been proposed. Different experimental evidence suggests that the loss of the GCase function in patient neurons, as well as in cellular or animal GBA models, compromises lysosomal protein degradation that in turn leads to an α-syn accumulation (for review, see [42]). In addition to their primary consequence—the lysosomal dysfunction—GBA1 mutations and abnormal GCase activity have also been linked to mitochondrial dysfunction [43,44]. Recently, a dysregulation of mitochondria–lysosome contacts in PD patient-derived dopaminergic neurons in the presence of reduced lysosomal GCase enzymatic activity was found, resulting in misregulated axonal distribution of mitochondria and decreased ATP levels [45]. Another suggested hypothesis is that GBA activity could modulate the cell-to-cell transmission/propagation of α-syn aggregates [46]. In this context, it is also noteworthy that GBA overexpression in vitro results in a significant decrease in exosome secretion of synuclein, while either the virus-mediated expression of mutant GBA in the mouse striatum or chronic inhibition of GCase activity in vivo result in an increase in exosome-associated synuclein oligomers [47].

Recently, another genetic link between aberrant lipid metabolism and PD has been identified. Mutations in the smpd1 gene, which encodes the lysosomal enzyme acid sphingomyelinase (ASMase), have been associated with an increased risk of PD [48,49,50,51]. Interestingly, ASMases with the L302P or fsP330 mutations compared with WT or A487V variant failed to reach the lysosomal compartment and were retained in the ER in transfected HeLa cells [51]. Moreover, in silico analysis suggests that mutations in SMPD1 disrupt either enzymatic domain fold or lipid-binding site [51]. Regardless of the SMPD1 molecular mechanism of toxicity, a reduction in ASMase activity by RNA interference approach or CRISPR/Cas9-mediated knockout both in HeLa cells and in the BE(2)-M17 dopaminergic cell line leads to an increase in α-syn levels likely due to an impairment in α-syn degradation by the lysosomal compartment [51].

### 2.3. Leucine-Rich Repeat Kinase 2 (LRRK2/PARK8)

Lrrk2 gene is the most frequently mutated gene in both sporadic and familial Parkinson’s disease (PD) cases, reaching up to 40% in some ethnic groups, Ashkenazi Jewish and North African Arab Berbers [52,53]. LRRK2 consists of four protein–protein interaction domains (armadillo repeats, ankyrin repeats, leucine-rich repeats and WD40 domain) and two catalytic domains (Ras of complex (Roc) domain associated with C-terminal of Roc (COR) domain and the kinase domain) [53]. Pathological LRRK2 mutations are autosomal dominant and are all distributed in the two catalytic domains including the most common mutation associated with LRRK2 (G2019S). Increased LRRK2 kinase activity has been proposed to strongly contribute to pathogenesis, suggesting the potential therapeutic use of LRRK2 kinase inhibitors in the treatment of PD [54]. However, LRRK2 pathological mechanisms of toxicity are still debated. The most prominent hypothesis is a direct LRRK2 involvement in the control of vesicle trafficking, strongly supported by LRRK2 association with intracellular membranes of different compartments, such as the Golgi complex, late endosomes, lysosomes, and synaptic vesicles and by the fact that many putative LRRK2 interactors belong to protein families involved in the regulation of vesicle trafficking or in cytoskeleton dynamics that in turn may modulate vesicle trafficking [53]. In neurons, the vesicle trafficking controls fundamental physiological functions, such as neurotransmitter or protein release and uptake, localization of membrane receptors, organelle biogenesis, and also changes in lipid membrane composition. Although an extensive analysis of lipidome profile in LRRK2 cellular or animal models is still missing, different lines of evidence suggest a potential role of LRRK2 in lipid metabolism and/or lipid signaling pathway(s). For instance, LRRK2 knockout mice show increased number and density of lipid droplets in both hepatocytes and stellate cells, compared with WT animals [55], and distinct lipid profile alterations [56]. In the same animal model, higher level of cholesterol was observed [55]. LRRK2 involvement in lipid metabolism was further confirmed in HepG2 cells, where the overexpression of LRRK2 promotes the β-oxidation by positively regulating carnitine palmitoyltransferase 1A, whereas LRRK2 knockdown inhibited β-oxidation [57]. Using a targeted lipidomic approach, Ferrazza and colleagues reported an altered sphingolipid composition in LRRK2 KO mice. In particular, ceramide content is significantly higher in KO compared with WT mice, suggesting that the absence of LRRK2 has an impact on ceramide metabolism [58]. Interestingly, ceramide is a component of all major sphingolipid species, and several studies have provided evidence that sphingolipid levels are often altered in neurodegenerative diseases, including PD [59,60].

LRRK2 may also play a role in the phosphoinositide metabolism. LRRK2 and synaptojanin1 (SYNJ1) loss of function share a similar pathogenic pathway in deregulating SV endocytosis in the dopaminergic neurons [58]. In particular, the SV endocytosis impairment seems to be mediated by direct LRRK2-mediated phosphorylation of SYNJ1, at least in vitro [61]. Interestingly, the results were further confirmed in drosophila models, where LRRK2 R1441C expression induces an enhanced phosphorylation of different SV proteins, including SYNJ1, in the brain [62]. SYNJ1 is a phosphoinositide phosphatase highly expressed in nerve terminals and able to dephosphorylate phosphatidylinositol bis- or trisphosphates, localized on plasma membranes. Inositol lipids are essential components of eukaryotic membranes and important intracellular second messengers activating different downstream pathways.

Numerous experimental results link LRRK2 and its phosphorylation substrates Rabs to lipids. Phosphoinositides and Rab GTPases regulate each other’s localization, and, importantly, membrane trafficking relies on dynamic changes in membrane identities that are determined by the regulation of distinct RAB GTPases and phosphoinositides. In fact, phosphoinositides mediate the recruitment of different Rab GTPases regulators, and Rab GTPases affect the recruitment of phosphoinositide regulators (for extensive review, see [63]). In some cases, the Rab binding to the membrane, in addition to prenylation, is dependent on a specific protein–protein interaction or on the presence of particular phoshoinosites [64]. For instance, the plasma membrane localization of Rab35 involves direct binding to the negatively charged phosphoinositides PtdIns(4,5)P2 and PtdIns(3,4,5)P3. Moreover, the molecular mechanism by which Rab35, a specific LRRK2 substrate [65], regulates different cellular functions, including endosomal trafficking, phagocytosis, cell migration and neurite outgrowth, probably involves regulation of phosphoinositides and F-actin, both on endosomes and at the plasma membrane [66]. Rab35 also controls the lipid turnover by myotubularins to repress mTORC1 activity and to control myelin growth [67]. Interestingly, the Rab proteins (including specific LRRK2 substrates) are also involved in lipid droplet (LD) formation and mobilization [68], and in fact, mutant LRRK2 Y1699C, by Rab8a phosphorylation on serine residue 72, regulates the fusion and enlargement of lipid droplets [69]. LDs consist of an organic core comprising neutral lipids (mainly triacylglycerols and sterol esters) bounded by a monolayer of phospholipids. Neutral lipids in LDs are mobilized by lipases to provide metabolic energy (through the oxidation of fatty acids) and lipids for membrane synthesis. Rab5 is associated to LD and modulates LD formation [64]. Rab10 is involved in the selective targeting of LDs to autophagic machinery by a Rab10/Dynamin-2 complex formation [70]. As previously mentioned, phosphorylation of Rab8A by LRRk2 promotes the formation of large lipid droplets [69] and Rab8A is required for muscle lipid uptake and storage [71]. Finally, Rab18 (although not specifically investigated as a LRRK2 substrate but showing a phosphorylation sequence similar to the other Rab members [54]) is a key regulator of LD formation and mobilization [72]. Interestingly, increasing evidence indicates that LDs dynamically interact with different cellular organelles, including mitochondria, endosomes, peroxisomes, and the plasma membrane and that this association might facilitate the exchange of lipids, either for anabolic growth of LDs or for their catabolic breakdown.

### 2.4. PTEN-Induced Kinase 1 (PINK-1/PARK6) and PARKIN (PARK2)

Although PINK-1 and PARKIN are considered key factors in mitochondrial quality control by promoting the removal of damaged mitochondria [73], different lines of evidence have implicated these two genes in the control of lipid and lipoprotein metabolism. Interestingly, lipid biology and mitochondrial homeostasis are tightly connected. Mitochondrial membrane lipids are essential for mitochondrial function. The biogenesis and the mitochondrial architecture, activity of respiratory protein, and transport of proteins into mitochondria are largely dependent on the mitochondrial lipid composition [74,75]. Valadas and colleagues, using both hypothalamic neurons differentiated from patient induced pluripotent stem cells (iPSCs) and fruit fly models lacking the parkin or pink-1 gene, found an excess of endoplasmic reticulum–mitochondria contacts [76]. These excessive contact sites cause abnormal lipid trafficking that depletes phosphatidylserine from the endoplasmic reticulum (ER) and disrupts the production of neuropeptide-containing vesicles without major defects in the mitochondria of mutant neuropeptidergic neurons. Importantly feeding mutant flies with phosphatidylserine rescues neuropeptidergic vesicle production and acutely restores normal sleep patterns [76]. Moreover, independent research groups have observed specific lipidomic alterations in mitochondria of aged PARKIN knock-out mice. For instance, Gaudioso et al. observed an enrichment in less unsaturated forms of CL, lower phosphatidylglycerol and phosphatidylinositol levels, and higher levels of some forms of hydroxylated ceramides [77]. Finally, a genome-wide RNAi screen performed in a PD cellular model to identify genes involved in PARKIN-mediated mitophagy identified the sterol regulatory element binding transcription factor 1 (SREBF1), a master regulator of lipid synthesis [78]. In SREBF1 knockdown, both Parkin translocation and mitophagy are altered [78]. Besides, the toxicity induced by PINK1 deficiency in different animal and cellular models was significantly reduced by the partial genetic or pharmacological inhibition of fatty acid synthase (FASN) [79]. Lower FASN activity in PINK1 mutants decreases palmitate levels and increases the levels of cardiolipin, a mitochondrial inner membrane-specific lipid. Furthermore, cardiolipin supplementation to isolated mitochondria rescues the PINK1-induced complex I defects and the inefficient electron transfer between complex I and ubiquinone [79]. The role of CL in mitochondrial physiology is of particular relevance because CL exposure to the outer membrane not only regulates mitophagy and the electron transport, but, relevantly for PD, also affects the α-synuclein aggregation [80]. Interestingly, in rotenone-treated rats, a reduction of polyunsaturated fatty acid (PUFA) cardiolipin and accumulation of mono-oxygenated cardiolipin species in the substantia nigra was observed, with an increase in PUFA-containing cardiolipins in the plasma [81].

Recently a new and specific role of parkin in fat intake and lipid metabolism has been described [82]. Parkin KO mice resisted weight gain, steatohepatitis, and insulin resistance when exposed to high-fat and -cholesterol diet [83]. The molecular mechanism of PARKIN effect seems to be the mono-ubiquitination of the class B scavenger receptor CD36 (also known as FA translocase, FAT), a transmembrane protein that binds with high affinity to a number of lipid ligands, including long chain FAs, anionic phospholipids, and native or modified lipoproteins [83].

### 2.5. Synaptojanin1 (SYNJ1/PARK20)

Synj1 has been recently identified independently by two research groups as the gene responsible of autosomal recessive, early-onset atypical parkinsonism (PARK20) [84,85]. Two main SYNJ1 isoforms, generated by alternative splicing, have been discovered of, respectively, 145 kDa (short isoform) and 170 kDa (long isoform). The short isoform was the first to be identified and it is highly expressed in the brain with a significant localization in the presynaptic nerve terminals [86]. SYNJ1 is a polyphosphoinositide phosphatase acting on various phosphoinositides, including phosphatidylinositol 4-phosphate, phosphatidylinositol (4,5)-bisphosphate (PI(4,5)P2), and phosphatidylinositol (3,4,5)-trisphosphate. Phosphoinositides are key regulators of cell physiology. In particular, PI(4,5)P2 plays a major regulatory role at the cell surface, both as a precursor of important signaling molecules, as well as via interactions with cytosolic and membrane proteins. The SYNJ1 protein contains two different phosphatase domains (N-terminal Sac1-like inositol domain and a central 5′-phosphatase domain) followed by a C-terminal proline-rich domain. The long isoform contains an additional proline-rich domain. Interestingly, most of the pathological SYNJ1 mutations are located in one of the two phosphatase domains, although recently, a new pathological SYNJ1 mutation was reported in the C-terminal domain of the longer isoform in a Tunisian family with juvenile Parkinson’s disease associated with epilepsy [87]. Up to now, by regulating phospholipid signaling, SYNJ1 seems to be mainly involved in the regulation of vesicle trafficking. For instance, synapses of knock-in mice carrying the homozygous R258Q mutation display endocytic defects and a striking accumulation of clathrin-coated intermediates [88]. Moreover, the pathological SYNJ1 mutation in the C-terminal domain, reported in the Tunisian family, is located in the clathrin adaptor protein 2 (AP2) binding domain, further supporting the SYNJ1 role the regulation of endocytic vesicle recycling in neurons [87]. Interestingly, as previously mentioned, LRRK2 directly phosphorylates synaptojanin1 in vitro, resulting in the disruption of endophilin–synaptojanin1 interaction required for SV endocytosis. Moreover, midbrain neurons from mice carrying both LRRK2 G2019S and SYNJ1+/− show a significant impairment in the exocytosis processes, strongly suggesting that LRRK2 and SYNJ1 control vesicle trafficking via a common pathological pathway [61]. The LRRK2–SYNJ1 physiological interaction was further confirmed in drosophila models, where the pathological LRRK2 R1441C mutant expression induces an enhanced phosphorylation of SYNJ1, both in vivo and in vitro [62].

### 2.6. Phospholipase A2 Group VI (PLA2G6/PARK14)

Mutations in PLA2G6 were first associated to neurodegenerative disease in the 2006. In particular, mutations in PLA2G6 were identified in a locus for infantile neuroaxonal dystrophy (INAD) and neurodegeneration with brain iron accumulation (NBIA) [89]. Independently, the genetic association was validated by Khateeb et al. in two consanguineous Israeli Bedouin kindreds with INAD [90]. They identified a 3 bp deletion in the homozygous PLA2G6 gene, leading to a valine deletion in position 691. Neurodegenerative disorders with high brain iron include Parkinson disease, Alzheimer disease, and several childhood genetic disorders categorized as neuroaxonal dystrophies. PLA2G6 was first associated to PD in 2009 by mutational analysis in three individuals from two unrelated families with adult-onset dystonia-parkinsonism (PARK14), and, surprisingly, none of the affected patients showed brain iron accumulation [91]. To date, different types of pathological mutations have been identified, including nonsense and missense mutations, small exons deletions, splicing sites. Patients with homozygous mutations in PLA2G6 show young onset, progressive cognitive decline, and dopa-responsive dystonia-parkinsonism.

The phospholipase A2 (PLA2) superfamily consists of many different groups of enzymes that catalyze the hydrolysis of the sn-2 ester bond in a variety of different phospholipids, leading to the release of arachidonic acid and other fatty acids [92]. PLA2 plays a key role in both phospholipid remodeling and signal transduction as the PLA2 products are important second messengers that play relevant roles in different signal transduction pathways. PLA2G6 encodes a calcium-independent group VI phospholipase A2, which localizes to the neuronal mitochondria and endosomal and lysosomal membranes. In the nervous system, PLA2G6 could be essential for the remodeling of membrane phospholipids in axons and synapses; however, the exact molecular mechanism by which PLA2G6 contributes to neurodegeneration has not yet been fully elucidated. For instance, in contrast to INAD mutations, it remains debated whether PD-associated mutations in PLA2G6 protein affect its catalytic activity [93].

Different PLA2G6 knock-out mice have been generated [94,95]. Malik et al. show age-dependent accumulation of distinctive spheroids in distal axons that contain membranes accumulated due to an impairment in axonal membrane homeostasis and in protein degradation pathways [96]. In old PLA2G6 knock-out mice, a significant neuroaxonal dystrophy was visible, likely due to insufficient remodeling and degeneration of mitochondrial inner membranes and presynaptic membranes [97]. The insufficient membrane remodeling was further confirmed by imaging mass spectrometry showing a significant increase in docosahexaenoic acid-containing phosphatidylcholine in the gray matter of the spinal cord of PLA2G6 KO mice, especially in the posterior horn [97]. Moreover, PLA2G6 KO mice showed decreased rates of incorporation of unesterified docosahexaenoic acid (DHA) from plasma into brain phospholipids, reduced concentrations of several fatty acids (including DHA) esterified in ethanolamine- and serine-glycerophospholipids, and increased lysophospholipid fatty acid concentrations [98].

In drosophila, PLA2G6 loss results in acyl-chain shortening in phospholipids, which affects ER homeostasis and neurotransmission and promotes α-synuclein aggregation. Interestingly, administration of linoleic acid or the overexpression of C19orf12, another NBIA-causative gene, rescues the acyl-chain shortening due to PLA2G6 loss [99].

## 3. Conclusions

**Figure 1:**
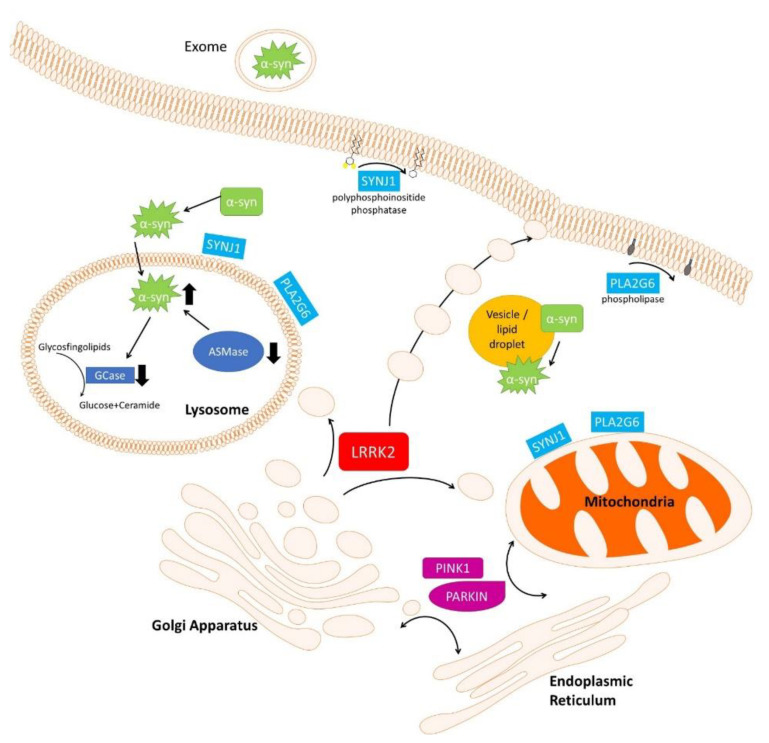
Cell biological processes impacted by PD-related genes leading to lipid alteration. As described in the main text, different PD-causative or -risk genes (GBA, SMPD1, SYNJ1, and PLA2G6) directly control lipid metabolism in neuronal cells. PD-causative genes (LRRK2, PINK-1, and PARKIN) may indirectly control lipid metabolism, localization or signaling by controlling vesicle trafficking and/or lipid exchange between various organelles inside the cells. Finally, α-synuclein physiology is tightly related to lipid: α-synuclein controls lipid metabolism, α-synuclein localization and aggregation are strongly dependent on a specific membrane lipid composition, and, lastly, LBs are largely composed of α-synuclein tightly associated to lipids and membrane fragments. Aggregated α-synuclein is represented as green stars.

There is a growing body of evidence linking dysfunctional lipid metabolism to PD pathogenesis. Changes in membrane lipids have been observed in both affected and unaffected regions of brains from PD patients, and in different experimental models expressing PD-causative or -risk genes, indicating that alteration in lipid metabolism/pathways may precede PD development. In Figure 1, some molecular pathways altered by PD-related gene expression leading to alteration in lipid metabolism, composition, or signal transduction are schematized.

Both α-synuclein localization and aggregation are dependent on specific membrane lipid composition. Moreover, lipid metabolism alteration in lysosomes for instance by GBA or SMPD1 mutants may affect the α-synuclein clearance. LRRK2 may regulate lipid metabolism either directly or, more likely, indirectly through the control of vesicle trafficking by the phosphorylation of different RAB family proteins. Eukaryotic cells rely on a complex and regulated network of vesicular transport to ensure efficient delivery of lipids to target organelles. Moreover, the endogenous lipid synthesis and delivery is of particular relevance in neurons because some circulating plasma lipids (e.g., cholesterol) cannot reach these cells due to the inability of different lipoproteins to traverse the blood–brain barrier. SYNJ1 is a polyphosphoinositide phosphatase acting on various phosphoinositides, and PLA2G6 is a calcium-independent group VI phospholipase A2; therefore. both are directly involved in the control of lipid metabolism. SYNJ1 and PLA2G6 are both localized on neuronal mitochondria, endosomal and lysosomal membranes, and plasma membrane and could play an essential role in remodeling membrane phospholipids either in cellular organelles or in axons and synapses. Importantly, the biogenesis and architecture of different cell structures (mitochondria, lysosomes, vesicles or lipid droplets, cell membranes) are largely dependent on the lipid composition. Furthermore, products generated by SYNJ1 or PLA2G6 are important second messengers that play relevant roles in different signal transduction pathways in neurons. Recent evidence has highlighted specific alteration in mitochondria due to abnormal lipid trafficking from the endoplasmic reticulum in both human hypothalamic neurons and fruit fly models lacking parkin or pink-1 genes. These data are further corroborated by specific alterations in lipid metabolism/composition in different PARKIN or PINK1 experimental models. Finally, experimental results indicating that LBs are largely composed of lipids, membrane fragments, and membranous organelles, such as vesicles, further confirm the importance of lipid composition in PD pathology.

## Data Availability

Not applicable.
